# Potential stocks and increments of woody biomass in the European Union under different management and climate scenarios

**DOI:** 10.1186/1750-0680-8-2

**Published:** 2013-02-01

**Authors:** Georg E Kindermann, Stefan Schörghuber, Tapio Linkosalo, Anabel Sanchez, Werner Rammer, Rupert Seidl, Manfred J Lexer

**Affiliations:** 1 International Institute for Applied Systems Analysis (IIASA), , Schlossplatz 1, A-2361 Laxenburg, Austria; 2, University of Natural Resources and Life Sciences (BOKU), Gregor Mendel Straße 33, A-1180 Wien, Österreich; 3 The Finnish Forest Research Institute, , PL 18, FI-01301 Vantaa, Finland; 4 Centre for Ecological Research and Forestry Applications (CREAF), Edifici C Campus de Bellaterra (UAB), 08193 Cerdanyola del Vallès, Barcelona, Spain

## Abstract

**Background:**

Forests play an important role in the global carbon flow. They can store carbon and can also provide wood which can substitute other materials. In EU27 the standing biomass is steadily increasing. Increments and harvests seem to have reached a plateau between 2005 and 2010. One reason for reaching this plateau will be the circumstance that the forests are getting older. High ages have the advantage that they typical show high carbon concentration and the disadvantage that the increment rates are decreasing. It should be investigated how biomass stock, harvests and increments will develop under different climate scenarios and two management scenarios where one is forcing to store high biomass amounts in forests and the other tries to have high increment rates and much harvested wood.

**Results:**

A management which is maximising standing biomass will raise the stem wood carbon stocks from 30 tC/ha to 50 tC/ha until 2100. A management which is maximising increments will lower the stock to 20 tC/ha until 2100. The estimates for the climate scenarios A1b, B1 and E1 are different but there is much more effect by the management target than by the climate scenario. By maximising increments the harvests are 0.4 tC/ha/year higher than in the management which maximises the standing biomass. The increments until 2040 are close together but around 2100 the increments when maximising standing biomass are approximately 50 % lower than those when maximising increments. Cold regions will benefit from the climate changes in the climate scenarios by showing higher increments.

**Conclusions:**

The results of this study suggest that forest management should maximise increments, not stocks to be more efficient in sense of climate change mitigation. This is true especially for regions which have already high carbon stocks in forests, what is the case in many regions in Europe. During the time span 2010–2100 the forests of EU27 will absorb additional 1750 million tC if they are managed to maximise increments compared if they are managed to maximise standing biomass. Incentives which will increase the standing biomass beyond the increment optimal biomass should therefore be avoided. Mechanisms which will maximise increments and sustainable harvests need to be developed to have substantial amounts of wood which can be used as substitution of non sustainable materials.

## Background

Forests play an important role in the global carbon flow. They can be used to store carbon and can also provide wood which can substitute non sustainable fossil fuel based energy sources or used e.g. for construction and furniture. Carbon storage and wood production are possible at the same time but they are also competing. If the management target is set to produce as much wood as is sustainable possible, which can be used as construction wood or for biofuel, will result in forests which are younger and have less standing biomass than a management target which is maximising the standing biomass. Both targets will help in climate mitigation. One by storing carbon in the forest the other by substituting fossil materials and storing the carbon in the not used fossils.

Forests also need to adapt to climate changes. Climate change will lead to increment changes and shifts in competition between tree species [1]. One practical way to make this adaptation is to select during reforestation tree species suitable to the new site conditions. This adaptation needs one rotation time to change from one to another species. The rotation times in forest management systems maximising the standing biomass are much longer than those maximising increments. [2] showed that an increasing growth trend can be observed in most cases, apart from some specific sites in Europe. [3] give net annual increment for EU27 with 550.6 mill.m^3^ (1990), 597.8 mill.m^3^ (2000), 619.5 mill.m^3^ (2005) and 608.9 mill.m^3^ (2010). It looks like that the annual increments have reached a peak around the year 2005 especially if it is taken into account that the forest area is increasing from 146.1 mill.ha (1990), 152.8 mill.ha (2000), 154.7 mill.ha (2005) to 157.2 mill.ha (2010). This increment trend reversal can be caused by many reasons. One obvious reason will be found in the increasing share of old forests in EU27. Forests show a typical increment pattern over age [4]. Young forests show low increments per hectare and year which are increasing with age until a certain age where the increment is culminating and a further increasing age shows a decreasing increment. This pattern is e.g. site, species and stand density depending. The standing biomass is increasing with an increasing age until the age of the climax phase and will decline in the following disaggregation phase. Those growth and biomass developments can be interrupted by several disasters. [3] show that the carbon in forests (above and below ground) is increasing from 7 806 MtC (1990), 8 782 MtC (2000), 9 317 MtC (2005) to 9 901 MtC (2010). This trend of increasing carbon stock is not only caused by the increasing forest area. It is also caused by higher average carbon stocks per hectare. This trend does currently not show to reach a peak. A declining increment rate and a constantly increasing biomass stock can only be realised by a reduction in harvests, and exactly this has been reported by [3] with 325.5 mill.m^3^ (1990), 378.1 mill.m^3^ (2000), 408.5 mill.m^3^(2005) and 387.6 mill.m^3^ (2010). The harvest pattern is following the increment pattern. Assuming a balanced age–site distribution this correlation between increment and harvests seems perfect for sustainable forest management. But the gap between increments and harvests cause an increasing average age and biomass and at certain point a decreasing wood increment. [5] showed the development of this trend until 2030 with an estimated marginal increasing wood demand for the time span 2005–2030. They also showed that the estimated increments are slowly decreasing until 2030. Beside the age and stand density with the current species composition, the increment trend is also affected by site factors. In the past environment pollution will have lowered the site productivity. Nowadays a changing climate and CO_2_ concentration show influence.

Most of the climate mitigation literature so far has assessed the potential contribution of purposeful management of terrestrial ecosystem management in terms of delivery of carbon neutral biomass for energy production where the overall terrestrial sink was assumed to stay constant over time [6]. [7] and all yield tables and most forest growth models which have been produced and used in forestry since that time, showed that using forests as carbon storage and for biomass production at the same time can not maximise both. If a forest has high increments it has medium standing biomass, if it has high standing biomass it has low increments. Both management targets (maximise increment – maximise standing biomass) are competing against each other. To show the effect of climate and management, forest growth models can be used. They can show the effect of climate change on site and its productivity rate. They can also show the effect of different management targets. Here we use a combination of plot and large scale forest growth models and couple climate scenarios with one management scenario which is increasing biomass stock and another which is increasing the increment rates. This allows showing the effect on standing biomass, harvests and increments and giving a recommendation which of those management targets will be better in respect of climate mitigation.

## Results and discussion

**Figure 1:**
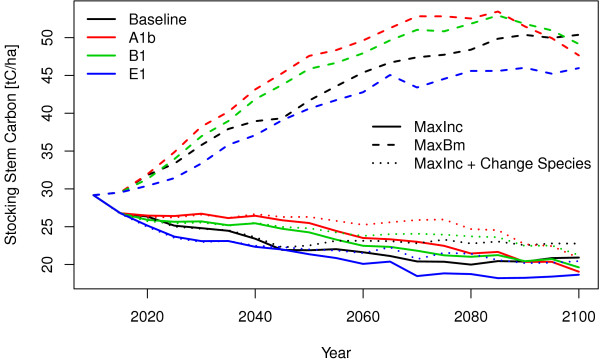
Stem carbon development under conditions of baseline and three climate change scenarios (A1b, B1 and E1) and three management scenarios (maximise stocking biomass, maximise increments with and without change of species).

Different climate and management scenarios show a typical development of the standing biomass over time. Figure 1 shows the development of the average standing stem carbon in EU27 assuming that the current forest area stays constant. The effect of the chosen management target has much more influence on the standing biomass than the different climate scenarios. The currently standing ≈30 tC/ha can be raised to ≈50 tC/ha within the next 60 years. The total forest area of EU27 is around 155 million hectares what makes a total carbon storage increase of around 3100 million tC until 2070 where the potential of carbon storage by choosing a long rotation time is reached. Of the climate scenarios E1 has the lowest potential. A1b and B1 show higher carbon storage potentials compared to the baseline but at the end of the century they loose some standing biomass and come very close together with the baseline scenario. The management which is maximising the increment decreases the currently standing stem biomass from ≈30 tC/ha to 20 tC/ha in 2100. This decrease is not caused by unsustainable usage or forest degradation. It is caused by the fact, that a forest, which is managed to produce optimal increments, has a certain stand density and also a certain rotation time which determines the standing biomass [8]. Starting from forests which have low stand density, very short rotation times or low standing biomass would show an increasing biomass with a increment optimising management regime. Reasons why the forest owners don’t use their increment potential can be that harvests of larger and older trees are most of the time cheaper [9], high harvest amounts will lower market prices of wood as it can be seen e.g. after large wind throws or the harvesting costs are higher than the wood price. Also the timber price depends on the tree dimensions. So an economic harvest decision will minimise harvesting costs and maximise timber prices by having high harvest amount. In this management scenario the E1 scenario shows also the lowest standing biomass and A1b and B1 are slightly above baseline but come in the year 2090 very close together. By allowing a change of tree species groups the standing biomass is at the end of this century about 3 tC/ha higher than the scenario that keeps the same species. In total this makes a carbon loss at around 1550 million tC until 2100. Comparing both management scenarios the one which is maximising biomass will store in 2100 approximately 4650 million tC more than the management-option that is maximising increments.

**Figure 2:**
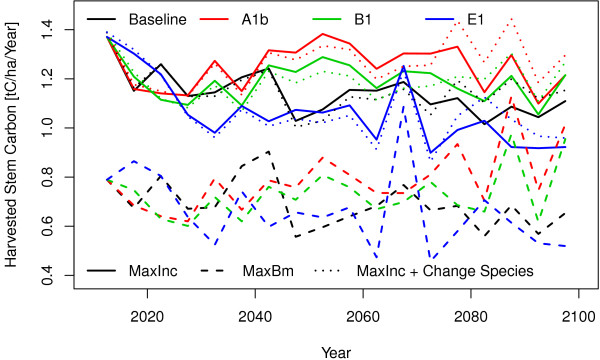
Removed stem wood under conditions of baseline and three climate change scenarios (A1b, B1 and E1) and three management scenarios (maximise stocking biomass, maximise increments with and without change of species).

Figure 2 shows the amount of removed stem wood per hectare and year. The amount of removed stem wood includes planned harvests but also unplanned mortality. Here the picture is contrary to the standing stem carbon (Figure 1) by having high amounts of harvest when maximising increment and low harvest amounts when maximising the standing stem carbon. By maximising increments harvests amounts are around 0.4 tC/ha/year higher than in the management that maximises the standing biomass. For B1 in the time span 2010–2100 this makes additional harvests of 40 tC/ha when maximising increment compared to maximising standing carbon. For EU27 in the time span 2010–2100 this makes in total 10 200 million tC stem wood harvests in the scenario maximising standing biomass but 16 600 million tC when maximising increments.

The scenario with and without species change show for this time range nearly the same values as the new planted species are mainly used by thinning. Some of those trees which are planted in the beginning of the simulation period can reach in the end of the simulation an age suitable for final harvest. The change from one to another species shows in the years until 2070 lower and afterwards higher harvest rates and it looks like that this new species are superior in the years after 2070. This indicates that decisions which are made today have a positive effect only in the long run. This is true especially for the species selection under changing environment.

If someone has to select a species today for regeneration someone can choose those species which are suitable for the current conditions or for the conditions expected in the future. The species selected for current conditions will be adapted to the current conditions but might have problems with future conditions, if selecting it for future conditions it might not survive until the time when the conditions fit. Also it is vague how the site conditions will be in the future. A risk reduction can be found in using several species for regeneration. They can be planted in pure stands but also in mixed forests. Mixed forests can but need not increase the increment rate compared to a pure stand [10,11]. If one species is suffering in a mixed stand the other species can make use the growing space of the suffering species and keep the total increment rate on a high level. In mixed stands one species can e.g. increase the water stress of another species [12] and also the regulation on the competition on light will need an increased management effort compared with pure stands. The species selection has also an effect on rotation time. Assuming there are two species showing the same potential of increment and are in all other concerns equivalent but have a different growth pattern which one should be preferred. To make it more illustrative let’s say one species is robinia, the other one oak. Oak shows in young ages low increments and is increasing slowly but is keeping the increment rate in old ages on a high level what results in applying long rotation times. Robinia shows a fast raise of increments in young ages but also a fast decrease in older ages what results in applying short rotation times. The long rotation times will cause high standing biomass but causes also a long time to change from one species to another. The short rotation will show the opposite. A recommendation in species selection will not be easy but a combination of short and long rotation will increase the number of species what spreads the risk. A combination of short and long rotation in the same stand can be realised with middle forest (coppice with standards), plenter and femel system but not with clear cut. Those silviculture systems will at least increase the management and harvesting costs and can cause different wood qualities. Using forests in very short rotation will have the disadvantages that the size of the trees might be small and can not be used as sawn wood what causes low wood prices. Also the carbon storage and the increments are reduced. A reduction of biomass opens the possibility to use for a short time more wood than is growing again. This amount of harvests will be increased for a short time in many regions of EU27 if the rotation time is reduced to an increment optimal rotation time for current species. If the replanted species have a shorter increment optimal rotation time and with this a lower standing biomass, this short time harvest amount is further increased. This opens the possibility to substitute now more fossil fuels and develop in the meanwhile efficient technologies which allow a reduction of energy use int the future. These increased harvests must not exceed the point where an additional reduction of the standing biomass causes a reduction of forest increment.

In E1 harvest shows a peak near 2070 which is caused by increased mortality caused by a climate situation which stresses the trees. It can also be seen, that the forests which are maximising biomass show more mortality (peaks in harvest amount) than those maximising increments. [3] shows also an increasing damage from 1990 to 2005. On the one hand this higher mortality is caused by the higher standing biomass on the other hand by the increased sensitivity of older trees against stress. If the damaged wood remains in the stand, the carbon will be stored there for some time, and especially wood of larger dimensions tend to decompose much slower than fine litter and will also increase the soil carbon. On the other hand this damaged and left wood can not be used for substitution and can increase the risk that the remaining trees are also damaged. In scenario A1b and B1 the harvests since 2050 are slowly decreasing when maximising increments and keeping the same species but they are increasing when maximising biomass. This is caused by a decreasing site suitability of the trees. This reduces the increments and so the model reduces the harvests but this reduction is overcompensated by mortality in the scenario which is maximising biomass. It can also bee seen, that the level of harvests could be more or less held constant during the simulation period in the management scenario which changes tress species. The deviation in the baseline scenario is caused by a climate pattern and the age structure of the forests. Comparing both management scenarios the one which is maximising increment will harvest in the time span 2010–2100 approximately 6400 million tC more than the management which is maximising biomass.

**Figure 3:**
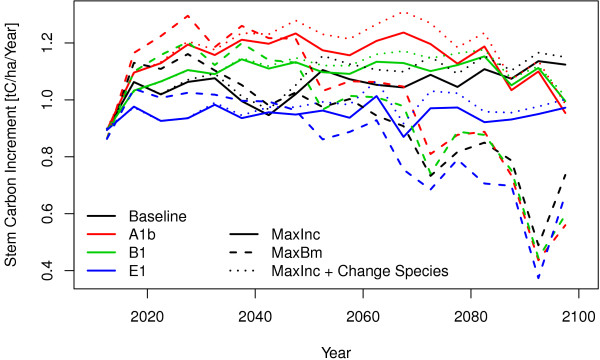
Increment development under conditions of baseline and three climate change scenarios (A1b, B1 and E1) and three management scenarios (maximise stocking biomass, maximise increments with and without change of species).

To complete the picture the increment for the climate and management scenarios are given in Figure 3 which need to be consistent with the already shown standing stem wood and removals in the respect that a change in the standing biomass is equivalent to the difference of increments and removals. In the first years there is not too much difference between the two management scenarios as a complete change of forest structure and age distribution needs typical one rotation time which is in many case 100 years and more. In the beginning the management which maximises the standing biomass shows higher increments than the one which maximises increments. On a first look this sounds counter intuitive. The reason for this is that the model does not maximize the increments in a short run. It starts to decrease the rotation time by increasing the harvests. Heavy harvests produce large areas which needs to be afforested and those young stands obviously show a lower productivity than the old stands which have been there previously. But the growth curves show that this short time disadvantage is turning some years later into higher increments than those that the old trees would be able to produce. The increments of the biomass maximising management is decreasing over the time. That means that the capacity of sequestrating carbon is getting lost with this strategy. For sure this capacity of sequestrating carbon can be increased again but this will need time and the lost increments can never be caught up. Changing to productive species will further increase the increment potential in the long run. Also in the baseline run the increment could be increased by changing the species.

**Figure 4:**
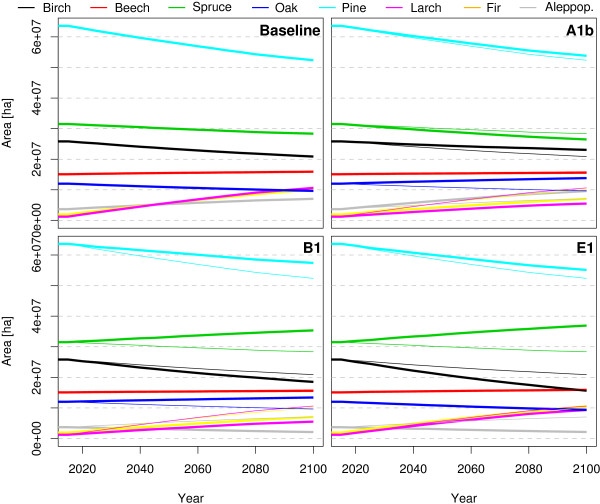
**Development of the cover area of the species groups Birch, Beech, Spruce,Oak, Pine, Larch, Fir and Aleppo pine when maximising increments and select species with high increments for reforestation with the climate change scenarios A1b, B1 and E1.** The thin line shows the area development of the baseline scenario.

**Figure 5:**
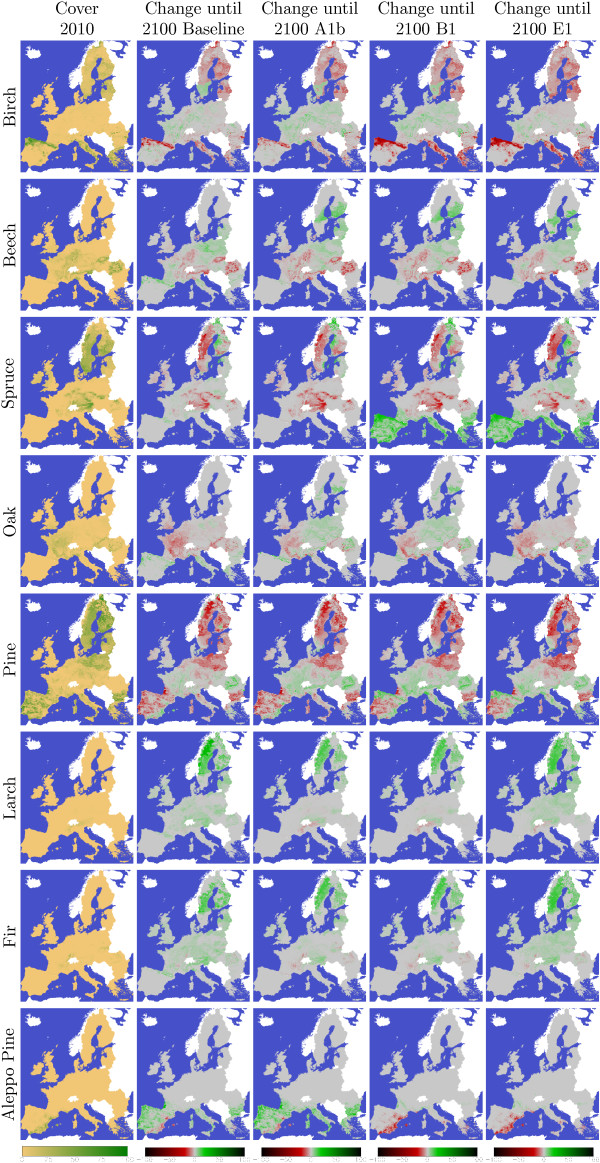
**Changes in species distribution.** The pictures in the left row show the current species distribution. The following 4 pictures show the change of the species share until 2100 for the four climate scenarios.

Figure 4 shows how the area of the 8 species groups is developing in the scenario which is maximising increments and selecting species with high increments for reforestation with the four climate scenarios. Figure 5 shows where the species are located and the regions of their changes until 2100 under the different climate scenarios. From the total forest area of 155 million hectare 16.7 % (25.8 mill. ha) is covered by Birch, 9.74 % (15.1 mill. ha) by Beech, 20.33 % (31.5 mill. ha) by Spruce, 7.75 % (12.0 mill. ha) by Oak, 41.1 % (63.6 mill. ha) by Pine, 0.79 % (1.2 mill. ha) by Larch, 1.25 % (1.9 mill. ha) by Fir and 2.38 % (3.7 mill. ha) by Aleppo Pine in the year 2010. Until the year 2100 the total area of Birch is reduced in all scenarios. The area is changed by -4.95 mill. ha (Baseline), -2.77 mill. ha (A1b), -7.33 mill. ha (B1), -10.19 mill. ha (E1) where birch looses area in the north and south and gains some share in central Europe. Beech is increasing its area by 0.82 mill. ha (Baseline), 0.51 mill. ha (A1b), 0.50 mill. ha (B1) and 0.82 mill. ha (E1). Beech loses partly in central Europe and gains in northern Spain and north east Europe. Spruce is changing its area by -3.15 mill. ha (Baseline), -5.06 mill. ha (A1b), +3.83 mill. ha (B1) and +5.42 mill. ha (E1). Spruce looses in central and north Europe and gains in the very north regions. In the scenarios B1 and E1 it also gains areas in south Europe. Oak changes its area by -2.38 mill. ha (Baseline), +1.82 mill. ha (A1b), +1.38 mill. ha (B1) and -2.66 mill. ha (E1). In Baseline it looses in central Europe and gains in south Europe. In A1b and B1 Oak looses in western Europe and gains in central, east and north Europe. The most present species group pine losses -11.26 mill. ha (Baseline), -9.80 mill. ha (A1b), -6.20 mill. ha (B1) and -8.51 mill. ha (E1). Pine looses in north and south and gains in central Europe. Larch is increasing its area by 9.39 mill. ha (Baseline), 4.27 mill. ha (A1b), 4.27 mill. ha (B1) and 8.10 mill. ha (E1). Larch has very small areas in 2010 and is loosing a little bit of its share in the alpine region and gains areas in north Europe. Fir is increasing its area by 8.15 mill. ha (Baseline), 5.10 mill. ha (A1b), 5.10 mill. ha (B1) and 8.55 mill. ha (E1). Also Fir has small forest areas and is loosing only very few of them. Fir can increase little in central Europe and substantial in north Europe. Aleppo Pine is changing its area by +3.37 mill. ha (Baseline), +5.91 mill. ha (A1b), -1.57 mill. ha (B1) and -1.54 mill. ha (E1). In all scenarios it losses area in south east Spain. In Baseline and A1b it can gain area in the rest of southern Europe. In B1 and E1 the yields for Aleppo Pine are taken from the species group Pine.

The shown changes of species should not be taken too seriously as the behaviour of trees in a changing environment is not perfect known and forest management can select other species than the model has selected. Also the used species groups are just a few of the huge amount of possible species. Some results look quite not very realistic like the area increase of spruce in the southern region in B1 and E1. Comparing the species shift with the estimations in [13,14] it looks like that predicting the tree species composition is uncertain as [13] does not show large changes until 2071–2100 in the A2 scenario but [14] shows huge changes until 2070-2100 for the A1b scenario. For the application the species selection is one major point which needs to be supported by giving some advice. Some small scale plot level models can support this decision but in large scale it looks like that there is still a wide field of improvement. In addition species show a genetic heterogeneity and the provenance need also to be taken into consideration.

**Figure 6:**
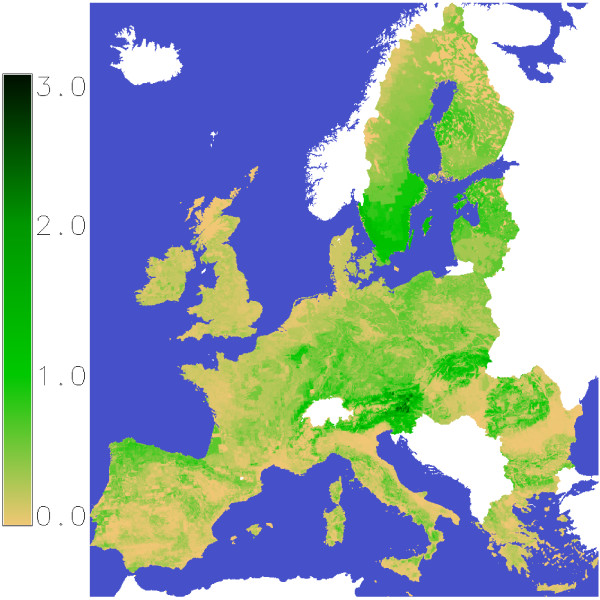
**Average stem wood increments in baseline scenario during the period 2010–2040.** Values are in tC/ha/Year × forest share.

Figure 6 shows the average increments for the time span 2010–2040 for the Baseline-scenario where the management is maximising increments and reforests the best growing species. Highest values can be observed in regions which have high forest cover and also high yields like in the Alp regions with lower altitude. In Sweden the effect of a decreasing yield from south to north can be observed.

**Figure 7:**
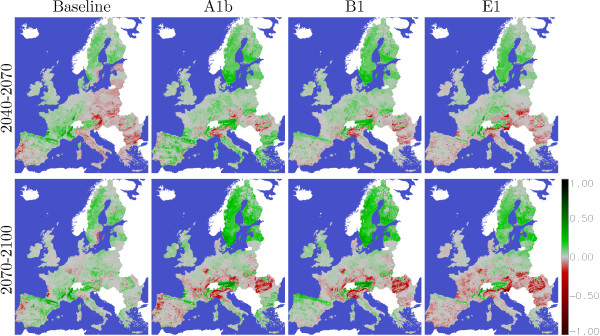
**Changes of stem wood increments for the time periods 2040–2070 and 2070–2100 and the four climate scenarios (Baseline, A1b, B1 and E1) shown as the difference to the increments of the time period 2010–2040 in Figure **6**.** Differences related to time period 2010-2040 in tC/ha/Year × forest share.

Figure 7 shows regions which can improve their increments and regions with declining increments. The figures show the increment differences compared to the time span 2010–2040 for their climate scenario where the management is maximising increments and reforests the best growing species. Even though in the baseline everything is static there are changes in the species composition, the age structure and also in the rotation time which affects the estimated increments. During the period 2040–2070 increments in western and northern Europe are increasing and in eastern and south Europe decreasing. In the time period 2070-2100 the increments in most regions could be raised due to selecting optimal rotation times and species with high yields. Regions which show a decrease are caused by age structures which have now age classes with high increments over represented. Theses age and species effects are also present in the climate scenarios but here is in addition a change of the environment. In A1b many regions show higher increments in the time span 2040–2070 than in the 30 years before but in the time span 2070–2100 only cold regions show increased yields most other regions show a declining increment. B1 looks similar like A1b but some regions in Spain show an increased increment. E1 looks also similar to A1b but the increment increases in cold regions are not as high as in A1b and the increment reduction on the remaining regions are lower than in A1b.

## Conclusions

Stocks and increments of the woody biomass are much more sensitive to forest management targets than to the climate change scenarios.

Adaption of forests to climate change is done during regeneration by choosing appropriate species. To reduce risk, mixed forests should be established. Regeneration is done after a final cut and a final cut is done in European forestry typical in the range of every 100 years. This rotation time needs to be increased if forests are managed to store high carbon amounts what reduces the ability of adaptation during regeneration. The absolute change of temperature or precipitation will be larger during long rotation times than during short rotation times. Therefore tree species for storing high biomass in a changing environment need to have a wide ecophysiologial spectrum, what will limit the number of possible tree species.

A policy focusing on storing high amounts of carbon in forests has to increase the rotation time and this will reduce in the long run the annual wood increments what will e.g. reduce the substitution of fossil fuels. Low wood increments will diminish forests ability in climate change mitigation. The model results indicate, that in EU27 the standing wood biomass is higher than the expected biomass in forest which are managed to maximise increments. A strategy which is using forests for storing carbon by increasing their biomass stock could be economical attractive for a short time but this strategy will decrease the amount of carbon fixation in forest in the long run to zero. The estimates show that in total (standing biomass and harvests) maximising increments is absorbing 1750 million tC more in the time span 2010–2100 in EU27 than the management which is maximising the standing biomass. This suggests avoiding any incentive which will increase the standing biomass beyond the increment optimal biomass and hinder a decrease of the standing biomass in many regions of EU27.

## Methods

To examine the development of 

•standing stem carbon,

•increments and

**Figure 8:**
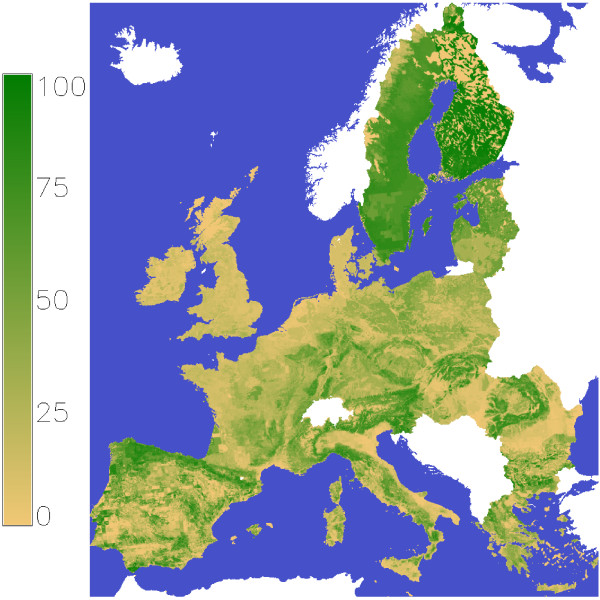
Forest cover map in EU27 in the year 2010.

•harvests

in the forests of the European Union (Figure 8) we used the global forest growth model (g4gm). The forest area and its location are not changed in the simulations, what will result in some underestimation as [3] showed a forest area increase for EU27 from 146 mill. ha in 1990 to 157 mill. ha in 2010 and this trend seems to continue. These three forest values of interest are influenced by site conditions and management. The site conditions are described by soil texture, slope, altitude, temperature and precipitation. The development of temperature and precipitation are estimated on a daily basis until 2100 by the REMO [15] model for the scenarios 

•Baseline,

•A1b,

•B1 and

•E1 (like A1b but limit to 450-ppm CO_2_ equivalent).

The plot level models Prelued, Picus and Gotilwa+ used the site information to estimate the potential productivity of forests. This productivity was used by the g4gm to predict the development of standing stem carbon, increments and harvests until the end of this century where forest management target is either to 

•maximise increments and keep current species

•maximise standing volume and keep current species

•maximise increments and select species with high increments for reforestation.

The scenario which changes species selects those species which have show at least 70 % of the average increment of the best growing species where the model looks 80 years into the future to make an increment ranking. The rotation time of all present species is set to the increment optimal rotation time but species who don’t reach these 70 % of increment their rotation time is limited to maximal 70 years what will result that these species are exchanged within the next 70 years.

### Aggregating to homogeneous response units

All data, beside the climate data, was available on a resolution of 1×1 km. The climate data was at a resolution of 25×25 km. This makes for the 27 member states of the European Union 4 304 383 grids on land with a size of 1 km^2^. Many of this grids show huge similarities in there site conditions. To reduce calculation time those grids have been merged to homogeneous groups. In the first step all grids which have the same 

•Country,

•Elevation category,

•Slope category and

•Soil texture.

are joined together to 2454 homogeneous groups. Where the categories are: 

•**Country:** Austria, Belgium, Bulgaria, Cyprus, Czech Republic, Denmark, Estonia, Finland, France, Germany, Greece, Hungary, Ireland, Italy, Latvia, Lithuania, Luxembourg, Malta, Netherlands, Poland, Portugal, Romania, Slovakia, Slovenia, Spain, Sweden, United Kingdom

•**Elevation category:** <300 m, 300–600 m, 600–1100 m, 1100–1600 m, 1600–2100 m and >2100 m

•**Slope category:** 0–3 %, 3–6 %, 6–10 %, 10–15 %, 15–30 %, 30–50 % and >50 %

•**Soil texture:** Coarse, Medium, Medium-fine, Fine, Very fine and Peat

In order to reduce the climatic variability within the individual response units, these 2454 regions have been split up into 14 168 sub-regions of similar climate. The splitting utilized k–means clustering based on the absolute variability of annual temperature, annual and summer precipitation, and temperature amplitude within response units. For each sub-region, average soil conditions were determined from the EU27 data grids.

**Figure 9:**
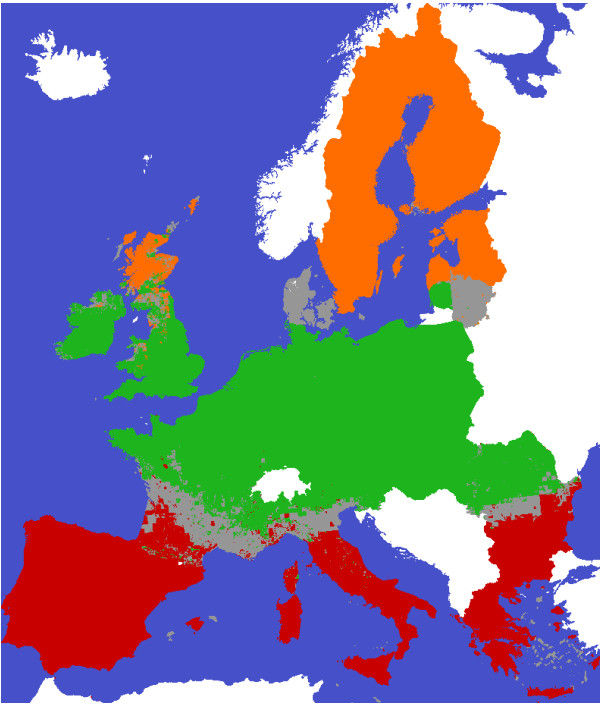
**The tree modelregions indicating the main application of the used plot level models.** Prelued was responsible for the northern (orange), Picus for the central (green) and Gotilwa+ for the southern (red) region. Adjoining models are responsible for the overlapping area (grey).

The area of EU27 was allocated to the responsibility of on specific plot level model. Figure 9 shows that Prelued was responsible for the northern (orange), Picus for the central (green) and Gotilwa+ for the southern (red) region. Adjoining models are responsible for the overlapping area (grey). This division was cancelled if one model was not able to make estimates of a specific species group (see Table 1).

**Table 1:** Modelregions

**Region**	**North**	**Central**	**South**
Birch	Prelued	Prelued	Prelued
Beech	Picus	Picus	Picus
Spruce	Prelued	Picus	Picus
Oak	Picus	Picus	Picus
Pine	Prelued	Picus	Gotilwa+^1^
Larch	Picus	Picus	Picus
Fir	Picus	Picus	Picus
Aleppo Pine	Gotilwa+^2^	Gotilwa+^2^	Gotilwa+^2^

^1^For B1 and E1 the estimates come from Picus.

^2^For B1 and E1 the values of Pine were used.

### Estimating yields

For each of the previously build homogeneous response units a yield level is estimated. As the site conditions are changing also this yield is changing from year to year. The yield estimates have been done by the tree plot level models: 

•**Prelued:** Yield estimates for the boreal zone were produced with a simple model of Gross Primary Production (GPP), described in detail in [16]. The model is based on light use efficiency (LUE) models [17], further modified by factors downscaling the photosynthesis due to low humidity (in air or soil), low temperatures or phenology. The potential GPP was calculated based on daily meteorological parameters, while soil water conditions were simulated with a simple dynamic model [16]. GPP was converted into NPP by subtracting the respiration as in [18] and equations from the “summary model” by [19] as well as yield tables from [20] where then used to produce the maximum Mean Annual Increment from the NPP estimates.

•**Picus:** The aim of PICUS3G is to provide a physiology–based, climate–sensitive estimate of forest NPP based on a parsimonious set of input data available at continental scales. PICUS3G is based on the production sub–module of the hybrid forest gap model PICUS v1.4 [21], and is a descendant of the generalized productivity model 3-PG [22]. GPP is calculated on a monthly time step using a light use efficiency approach. The fraction of the absorbed radiation in the canopy that is utilizable for photosynthesis is determined by limiting environmental factors, i.e., temperature, soil water availability, and vapor pressure deficit [21]. The quantum use efficiency is modified by the CO_2_ concentration in the atmosphere and by available nitrogen as a proxy for soil nutrient supply. NPP is derived from GPP using a constant respiration fraction, and is split into above- and belowground fractions based on environmental conditions, assuming that a more favourable environment result in a higher share of aboveground allocation [22]. PICUS3G requires plant-available nitrogen (kg/ha) and water holding capacity (mm) as well as temperature, precipitation, vapor pressure deficit, and radiation data at monthly resolution as model drivers.

•For the current contribution NPP was calculated for fully stocked, monospecific stands using the leaf area index (LAI) of even-aged stands at peak productivity. Simulations were conducted for beech, oak, fir, pine, spruce and larch (Table 1). G4gm used the NPP estimates generated by PICUS3G as a yield level indicator and scaling factor to adjust its empirically aggregated stand growth function. This model linkage thus allows an estimation of productivity based on physiological principles and environmental drivers (PICUS3G) while efficiently considering management in the subsequent application of the empirical model G4M.

•**Gotilwa+:** Results of the process-based forest growth model Gotilwa+ [23-27] are computed for DBH classes and integrated at the stand level. Although the present study has been carried out for even aged forests, most of Mediterranean forests are uneven aged. Information from National Forest Inventories do not include age as a measured variable and in fact some more detailed studies carried out in local forest inventories show that, in most cases, no relationship can be established between dominant tree height and age or between DBH and age in this water limited and with intense competition forest ecosystems [28]. That is part of the reason why Gotilwa+ does not include age as an explicit variable in the model. As g4gm model needed yield estimates at a determinate age of the forest, the Gotilwa+ model calculations were performed in such a way that the simulation started with a tree plantation and so the year of the simulation was assumed to be the age of the forest too. Climate files were reorganized in 50-year files with a 25-year overlapping, so that for each simulation unit there were 4 runs with an initial 25-year simulation to get a forest of 25 years of age and the rest of the results were used by g4gm to calculate the MAI. The output variable that was provided to g4gm was annual stem wood increment (tC/ha/year).

The g4gm model will use the mean annual stem-wood increment at increment optimal rotation time (MAI) in units of ton carbon per hectare and per year [tC/ha/year] as a yield descriptor. None of the three plot level model was able to provide their yield estimate in MAI. It was also not possible to find another yield description which all three plot level models could provide. So it was necessary to transfer three individual yield estimates with different definitions to one definition. For this task the growth functions of g4gm are used (equation 1) but the parameters to estimate the shape (factor *k* in equation 4 describes if increments in young or old ages are high or low), the highest age of increment (*t*_*max*_ equation 5) and maximum total carbon production of stemwood per hectare (TCP_*max*_ equation 6) are estimated individually for each plot level model and each species using several growth estimates from the plot level model itself for each of their species on a range of poor to very productive sites. The coefficients are in Table 2 where the growth curves for the Prelued model where calculated by using estimates form the PipeQual model [29] and a finish yield table [20]. The yield tables from [30,31] are used to compare Pinus sylvestris and Pinus halepensis with growth patterns from Gotilwa+.

**Table 2:** Growth curve coefficients estimated with growth data from the plot level models and yield tables

**Source**	**species**	***c***_**0**_	***c***_**1**_	***c***_**2**_	***c***_**3**_	***c***_**4**_	***c***_**5**_	***c***_**6**_	***c***_**7**_	**Figure**
PipeQual	Spruce	-0.2	-0.4	-0.1220	1.8345	350	100	0	0.776	10
PipeQual	Pine	-0.2	-0.5	-0.6361	1.1998	200	300	1.816	-1.246	10
Ilvessalo	Birch	-0.1	-0.5560	-0.1365	-2.0064	400	-325.070	4.985	-3.5	10
Picus	Beech	-0.2671	-0.2334	-3.8842	-1.8490	474.789	655.717	-7.068	4.402	11
Picus	Spruce	-0.3641	-52.7111	-6.1741	0.2943	-646.403	945.370	-1.643	-1.468	11
Picus	Oak	-0.1781	-1.0421	-3.7204	1.0574	300	1512.405	2.885	-3.722	11
Picus	Pine	-0.2523	-0.3219	-14.8589	-2.8190	261.892	323.775	-9.763	4.875	11
Picus	Larch	-2.936e-01	-4.020e-02	-2.855e+06	-1.247e+01	285.788	116.032	-17.287	5.248	11
Picus	Fir	-0.3449	-1.3433	-3.4040	0.6076	186.619	142.930	1.337	-3.258	11
Gotilwa+	Pine	1.246963	-1.097645	0.172259	-0.006605	3000	1400	2.875	-2	12
Gotilwa+	Aleppo Pine	-0.08	0.02212	-0.05967	-1.37455	2600	1200	1.491	-2	12
Abejon	Pine	0	-0.54	-0.3369	0.5073	225	65	16.69	-21.22	12
Montero	Aleppo Pine	-0.3	-0.306	-2.052	1.673	150	130	1.898	-1.141	12

**Figure 10:**
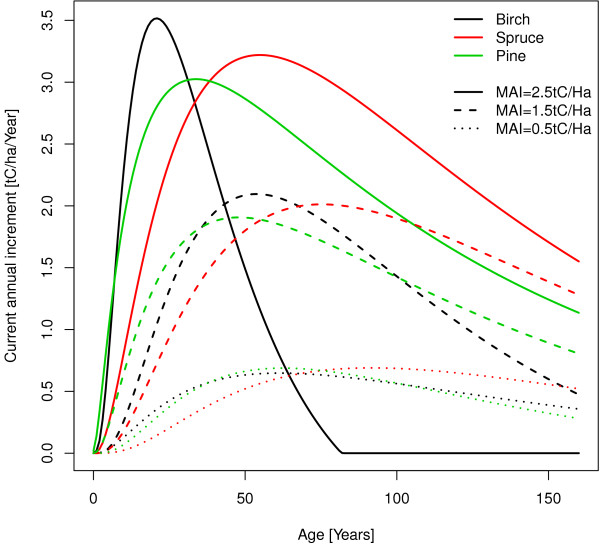
**Growth curves of the northern region form the PipeQual model **[29]** and a finish yield table **[20]**.**

The shapes of the growth curves of the northern region are shown in Figure 10. PipeQual [29] simulations (Pine and Spruce) were made from full stocked managed forests, the tables from [20] (Birch) are for natural forest. On productive sites Birch is culminating very fast followed by Pine and ending with Spruce. On average and on less productive sites Pine and Birch are showing similar patterns and spruce shows a slowly starting increment but the increment level is kept high in old stands. Species on productive sites show a distinctly earlier culmination than the low productive sites. Note that these curves don’t show the growth pattern of the species for a specific stand. They show the growth for the species if their yield level, expressed as the highest mean annual increment, is at a specific level and there is no need that this yield level is equal for different tree species on the same site.

**Figure 11:**
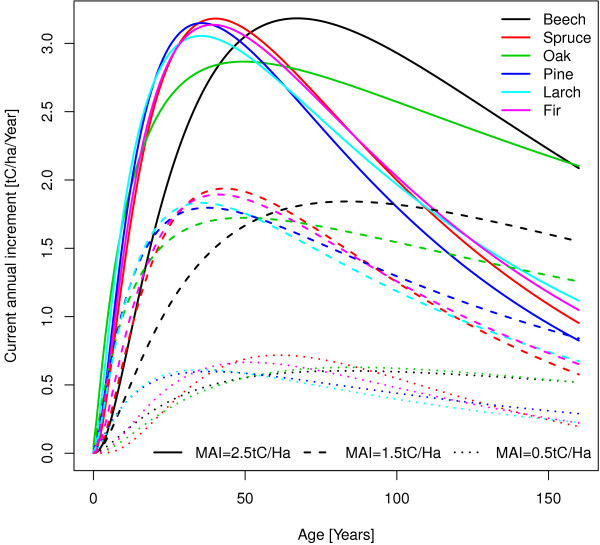
Growth curves of the central region from the Picus model.

The shapes of the growth curves of the central region are shown in Figure 11. On productive and average sites the growth pattern of coniferous trees are very similar. Oak and beech show a later culmination and afterwards a very slow growth decrease. On low productive sites Pine and Larch are similar and show an early, spruce and fir are similar and show an average and Oak and Beech are similar and show a late growth culmination. There are spares differences in the age of growth culmination between high and low productive sites.

**Figure 12:**
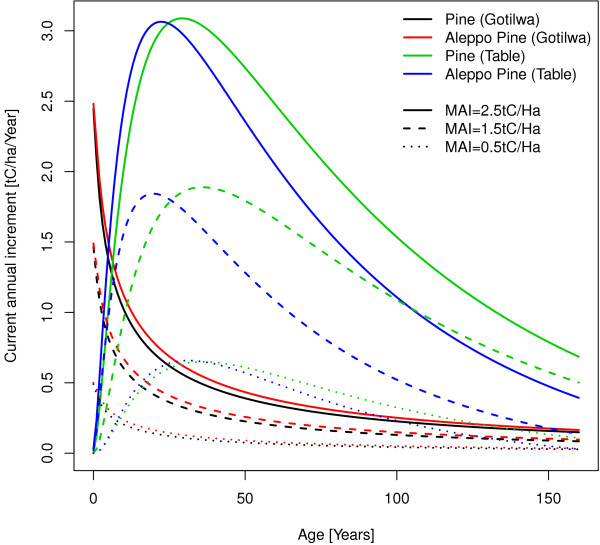
**Growth curves of the southern region from the from Gotilwa+ model and yield tables from **[30]**,**[31]**.**

The shapes of the growth curves in the southern region are shown in Figure 12. Those from Gotilwa+ look quite different compared to those of the other two plot level models. Yield tables from the southern region show similar growth patterns with the other plot level models but not like those from Gotilwa+. In Gotilwa+ the increment is decreasing monotonously when the age is increasing. This difference will not make to much problems as those growth curves from the regional plot models will only be used to convert their yield estimates to the yield estimate usable by g4gm. Growth curves like those from Gotilwa+ will recommend a rotation time of one year for forests to maximise the average increment what will not be to realistic. Also the total increment until a specific rotation time will show huge differences between the increments of Gotilwa+ and the local yield table for the same MAI.

How is this yield estimate conversion done? Prelued estimates the highest stem wood productivity in one year at a specific, but not given, age. For example it says that the highest stem wood increment of Birch is 3.5 tC/Ha/Year. With this information g4gm will have a look on the growth curves in Figure 10 and see that this maximum increment of 3.5 tC/Ha/Year are corresponding an MAI of 2.5 tC/Ha/Year. So this transformation is quite simple. There is just a need of one table showing the maximum increment and the corresponding MAI by using the proper growth curves.

Picus estimates the net primary productivity at an age of 50 Years (NPP50). The first step is to convert the NPP50 to stem wood increment also at age 50 in tC/ha/year. This is done by using estimates directly from Picus showing the ratio of how much of the NPP is stored in the stem. In Picus the assumption was made that this ratio is independent of site, age and productivity what will result in a species specific constant conversion factor. This factor will convert the NPP50, given in tC/ha/year, to stem wood increment, given in m^3^/ha/year (see Table 3). This factor includes beside the share of increment which is stored in the stem, a conversion from tC to m^3^ by using wood density and carbon content of wood. The carbon content of wood was assumed to be for all species 0.5 tC for 1 t dry woody biomass. The transformation from wood volume to weight typical is done by using the volume of wet wood and the weight of absolutely dry wood. The wood density *ρ*_0_ need to be corrected by the degree of wood shrinking. The wood density is varying between species, sites and others and is even not homogeneous within a tree. As the conversation was done internally in Picus from weight to volume there will not arise any error if the conversation from this estimated volume back to weight is done by using the same coefficient which can be found in Table 3.

**Table 3:** Coefficients from Picus to convert NPP to stem increment

**Species**	**Stem/NPP**	**Wood density*ρ***_**0**_
Beech	1.24579	680
Spruce	1.92055	430
Oak	1.24576	650
Pine	1.787301	490
Larch	1.64733	550
Fir	1.93735	430

NPP …net primary productivity at age of 50 Years [tC/ha/year], Stem …stem wood increment [m^3^/ha/year], Wood density …Wood density of oven dry wood [kg/m^3^].

After the first step converts the NPP50 to stem wood increment at age 50 in tC/ha/year this stem wood increments is converted into MAI by using the extracted growth curves from Picus. If e.g. the stem wood increment at age 50 is 2.8 tC/ha/year for Oak it can be seen in Figure 11 that this is equivalent to an MAI of 2.5 tC/ha/year. For this conversion there is a need for one table which shows the stem wood increment at age 50 and the MAI. Such a table can be created by using the growth curves. There might arise one problem during this conversion if there are two different MAI’s for one NPP50. In this case the lower MAI is used. Also it will be possible that there exists no MAI for a given NPP50 but this case did never appear in this study. Another problem is that the size and leave area of the trees at age 50, which is needed in Picus, for a specific yield is currently not known in advance so they were set to an average constant value. As long as the Picus model is not sensitive to tree sizes the expected bias in the predictions will be low.

Gotilwa+ estimates the stem wood increment at an increasing age with a tree size according to cumulative increments of the previous years. If the yield of the previous years is comparable the tree size will be correct, if there is a changing yield the tree size will not fit perfect to the age and yield combination. As long as the Gotilwa+ model is not very sensitive to tree sizes this will cause an acceptable bias in the predictions. The transformation from stem wood increment at a given age to MAI works the same way like for Prelued and for Picus by looking on the growth curves. E.g. if Gotilwa+ estimates an increment of 0.5 tC/ha/year for Pine with an age of 30 years it will be equivalent with an MAI of 2.5 tC/ha/year by making the conversion with the curves of Gitilwa+ in Figure 12. To make yield estimates Gotilwa+ plants trees and let them grow for 50 years. The values of the first 25 years are only used to create up forests with an age of 25 years. The following 25 years, where the forest has an age of 25 to 50 years, are used to estimate the yield. For the transformation to MAI 25 tables (one for each age class) for each species are needed which show the combination of stem wood increment and MAI.

The estimated yields of the climate scenarios are not identical for the starting year 2010. To let the simulations start at the same point the yield estimates for the first 5 year period are set to those of the baseline scenario.

### Creating initial forest conditions

After knowing the yields it is necessary to have realistic forest information where the simulation can start on. Therefore the following information will be needed: 

•Forest area

•Species share

•Age class distribution

In addition also the stand density of a forest will have an influence on the standing biomass and the increment. For this investigation the starting stand density is set to a yield table stocking degree of one.

The forest area was taken from the European Commission Joint Research Centre (JRC) Institute for Environment and Sustainability. There we select the forest cover map of the year 2006 where the map was indicated to be in the beta stadium. This map has a spatial resolution of 25×25 m. This map indicates that a grid is or is not covered by forest. A grid which is indicated to be covered by forest is treated as 100 % forest coverage and those which are indicated to be not covered by forest are treated to have 0 % forest cover. This map was aggregated to a 1×1 km map, which is congruent with the map which was used for the homogeneous response units, and gives now the information which share of this area is covered by forests. In the next step the tree species maps, also from the JRC Institute for Environment and Sustainability, which have a resolution of 1×1 km showing the share of the species in the year 2000 are used. These maps distinguish between 118 different species (groups). Here we will aggregate them into the following eight species groups: 

•**Beech:** Acer campestre, Acer monspessulanum, Acer opalus, Acer platanoides, Acer pseudoplatanus, Carpinus betulus, Carpinus orientalis, Castanea sativa (C. vesca), Eucalyptus sp., Fagus moesiaca, Fagus orientalis, Fagus sylvatica, Fraxinus angustifolia spp. oxycarpa (F. oxyphylla), Fraxinus excelsior, Fraxinus ornus, Ostrya carpinifolia, Platanus orientalis, Tilia cordata, Tilia platyphyllos, Ulmus glabra (U. scabra, U. scaba, U. montana), Ulmus laevis (U. effusa), Ulmus minor (U. campestris, U. carpinifolia), Arbutus unedo), Arbutus andrachne, Other broadleaves

•**Birch:** Alnus cordata, Alnus glutinosa, Alnus incana, Alnus viridis, Betula pendula, Betula pubescens, Buxus sempervirens, Corylus avellana, Ilex aquifolium, Populus alba, Populus canescens, Populus hybrides, Populus nigra, Populus tremula, Salix alba, Salix caprea, Salix cinerea, Salix eleagnos, Salix fragilis, Salix sp., Sorbus aria, Sorbus aucuparia, Sorbus domestica, Sorbus torminalis, Erica arborea, Erica scoparia, Erica manipuliflora, Phillyrea latifolia, Pistacia lentiscus, Pistacia terebinthus, Crataegus monogyna

•**Oak:** Juglans nigra, Juglans regia, Malus domestica, Olea europaea, Prunus avium, Prunus padus, Prunus serotina, Pyrus coomunis, Quercus cerris, Quercus coccifera (Q. calliprinos), Quercus faginea, Quercus frainetto (Q. conferta), Quercus fruticosa (Q. lusitanica), Quercus ilex, Quercus macrolepis (Q. aegilops), Quercus petraea, Quercus pubescens, Quercus pyrenaica (Q. toza), Quercus robur (Q. pedunculata), Quercus rotundifolia, Quercus rubra, Quercus suber, Quercus trojana, Robinia pseudoacacia, Ceratonia siliqua, Cercis siliquastrum

•**Aleppo Pine:** Pinus halepensis

•**Fir:** Abies alba Abies borisii-regis Abies cephalonica Abies grandis Taxus baccata

•**Larch:** Larix decidua Larix kaempferi (L.leptolepis)

•**Pine:** Cedrus atlantica, Cedrus deodara, Cupressus lusitanica, Cupressus sempervirens, Juniperus communis, Juniperus oxycedrus, Juniperus phoenicea, Juniperus thurifera, Pinus brutia, Pinus canariensis, Pinus cembra, Pinus contorta, Pinus leucodermis, Pinus mugo (P. montana), Pinus nigra, Pinus pinaster, Pinus pinea, Pinus radiata (P.insignis), Pinus strobus, Pinus sylvestris, Pinus uncinata

•**Spruce:** Picea abies (P. excelsa), Picea omorika, Picea sichensis, Pseudotsuga menziesii, Thuya sp., Tsuga sp., Other conifers

The aggregated species maps have been merged with the corresponding yield estimate form the plot level models. All grids where the yield estimate for a species in the years 2000-2010 show at least one year with zero increment are set to not occupied with this species. With this procedure the original area of birch was reduced by 1 %, beech 34 %, spruce 8 %, oak 56 %, pine 11 %, larch 23 %, fir 47 % and aleppo pine by 39 %. This reduction is partly compensated as the remaining species get the area of the removed species. On grids indicating they are covered by forest but there is now no species present are set to show no forest. This reduces the original total forest area by 6.7 %. The next task is to bring the total forest area of a country to the given values in [32]. This is done by summing up the forest area of the grids belonging to a certain country and transforming the forest share, which can have a range from 0 (no forest) to 1 (grid is full covered with forest), by an exponent. So the forest area is calculated with: $\text{forest area}=\sum (\text{gridSize}\times {\text{forestShare}}^{x})$ where the exponent x is chosen for each country individually that the calculated Forest area is equivalent with the forest area of the country statistics (Figure 8). In addition it is possible, that the origin forest shares are overlaid with a random deviation. This will help e. g. to affect also grids which show no forest cover or are full covered with forest and also will avoid that the distance between forest cover classes is changing so that some classes are over represented and others are empty. As we have currently 1600 forest cover classes with equal distance, an overlay of a random number was not done. The share of the species groups was adjusted the same way like the forest shares but the adjustment was not done for the species groups itself, instead it was done for broad leafed and needle trees. The country statistics of the broad leafed and needle trees shares are taken from [33] where the group mixed forests is added to the coniferous and broad leaved group where 25 %–75 % of the mixed forest area is added to broad leaved or coniferous choosing the share to have low shifts of the starting assumptions.

These forest area shares by different species can be divided into different age classes. Therefore the increment optimal rotation time (*t*_*opt*_) individually for each region and species using the average MAI for the years 2000–2010 is estimated using equation (2). This rotation time is increased depending on the slope by multiplying it with the following factors: 1 (slope 0–6 %), 1.04 (6–15 %), 1.37 (15–30 %), 1.61 (30–50 %), 1.13 (>50 %). E. g. when the increment optimal rotation time is estimated with 100 years and the forest is located on a site with a slope of 20 % the typical age of harvest is estimated with 137 years. These factors are estimated by using a laser scanner biomass map of Vorarlberg and take into account, that harvest is postponed if the slope is increasing. Until *t*_*opt*_× slope factor each age class has the same area share. Ages beyond *t*_*opt*_× slope factor get the right wing of normal distribution area share where the standard deviation of this normal distribution is calculated with 0.2×*t*_*opt*_× slope factor. These areas are summed up per country and age class separate for broad leaved and coniferous trees. With this you have an age class distribution which can be brought to those from [33]. The given area of the age class “unknown” in [33] is shared proportional to each known age class. The used age classes are 1–10, 11–20, 21–40, 41–60, 61–80, 81–100, 101–120, 121–140 and >140 years. The mixed forest age class area is divided according to the previous made division to broad leaved and needle trees. The previous created species groups are aggregated also into broad leaved and needle trees. To bring the initial estimated age structure to the observed age structure those estimates are increased or decreased with iterative modified multipliers. These multipliers for each age class needs to be applied for each region, slope class and species group (needle or broad leaved) and the total area needs to stay the same in each of theses groups after this multiplication. This means that if only one multiplier of one age class is changed also all other age classes are affected. Keeping the areas constant after multiplication is done by multiplying the new area of each age class with $\frac{\sum \text{Area}}{\sum \text{Area after multiplication}}$ where the sums are build for each region. The multipliers are iterative updated by setting $\text{new multiplier}=\text{old multiplier}\times \frac{\text{target age class share}}{\text{current age class share}}$. This allows creating starting conditions for the model which are close to given forest area, species distribution and age distribution from country statistics.

### Forest development estimates with the global forest growth model

#### Increment Functions

The increment functions are able to describe the (1) total carbon production of stemwood per hectare (TCP) at increment optimal stand density (SD_*opt*_) depending only on age, (2) estimate the managed stand density and (3) the maximum possible stand density, estimate (4) the tree size (DBH, height) and (5) the influence of stand density on TCP and DBH increment. All coefficients of the growth model are in Table 4. The coefficients are found by applying regressions on the yield tables of [31,34].

**Table 4:** **Coefficients of the growth model (equation (**1**)–(**12**))**

	**Pine**	**Beech**	**Birch**	**Fir**	**Larch**	**Oak**	**Spruce**	**P.Halep.**
*c*_0_	-0.3835	0	0	-0.4562	0	0	0	-0.3
*c*_1_	-0.2416	-0.5998	-0.7422	-0.7403	-0.388	-0.6	-0.9082	-0.306
*c*_2_	-1.7576	-0.2467	-0.54	-1.0772	-0.01226	-0.4419	-0.2728	-2.052
*c*_3_	1.1638	0.7674	0.5719	1.4803	0.8593	0.3179	0.6483	1.673
*c*_4_	170	245.6	137	0.6713	195.4	16.67	209.7	150
*c*_5_	114.3	100	100	300	600	300	300	130
*c*_6_	-2.804	2.6345	0.2972	-0.2151	0.9883	-0.6066	1.8536	1.898
*c*_7_	1.044	-0.8978	-0.7543	-0.9929	1.0784	-1.1243	0.4811	-1.141
*c*_8_	0	0.69135	0	0.5	0	0.7	0	0.92
*c*_9_	0.9	0	0.9	0.2	0.9	0.3	0.9	0.07
*c*_10_	-0.8242	0	-0.953	-0.7642	-2.1347	-0.4339	-0.143	-4.25
*c*_11_	-0.4273	0	-0.9236	0.3156	-0.3437	0.5288	-0.5915	6.168
*c*_12_	-0.4	-0.03177	-0.4	-0.4	-0.4	-0.4	-0.4	-0.4
*c*_13_	-1.476	0	1.052	0.4468	1.3238	2.0156	0.4507	0.9324
*c*_14_	4.283	0	0.108	0.1425	0.4061	-0.07354	0.3713	-0.00468
*c*_15_	-0.3	0	0	0	0	0	0	0
*c*_16_	3.61	0	0	0	0	0	0	0
*c*_17_	-1.071	0	0	0	0	0	0	0
*c*_18_	0.1	-0.875	0.1	0.25	0.1	0.1	0.1	0.25
*c*_19_	1.127	2	1.082	-0.865	1.082	1.03	1.135	-0.865
*c*_20_	-0.3028	0	-0.4	-0.5	-0.3	0.3	-0.3	-0.5
*c*_21_	0.5	0.5	0.5	0.5	0.5	0.5	0.5	0.5
*c*_22_	22.09	21.29	23.24	24.83	23.63	21.26	22.59	26.59
*c*_23_	0.6207	0.4872	0.4455	0.6071	0.5028	0.5199	0.6168	0.6284
*c*_24_	-0.0197	-0.0197	-0.0249	-0.0212	-0.0156	-0.019	-0.021	-0.0202
*c*_25_	1.5061	1.8148	1.3697	2.4131	1.162	1.3408	2.4176	1.0595
*c*_26_	-0.2535	-0.2914	-0.4294	-0.4825	-0.1867	-0.1098	-0.3582	-0.0349
*c*_27_	22.7	30.71	13.61	16.11	25.2	-7.511	16.11	18.73
*c*_28_	16.56	7.008	10.69	17.78	9.118	41.69	17.78	46.38
*c*_29_	-0.0107	-0.0105	-0.0269	-0.0144	-0.0138	-0.022	-0.0144	-0.2643
*c*_30_	0.248	-0.198	0.242	0.374	0.646	0.581	0.374	14.143
*c*_31_	-1.814	0.298	-0.702	-1.524	-0.799	1.725	-1.524	-0.637
*c*_32_	1.095	1.423	1.337	2.282	1.082	3.676	2.282	0.895
*c*_33_	0.1	1.025	0.071	1.272	0.167	1.754	1.272	0
*c*_34_	-1.603	-16.85	-2.151	-0.771	-0.941	0.326	-0.771	-4.963
*c*_35_	1.6	1.6	1.6	1.6	1.6	1.6	1.6	1.6
*c*_36_	1.5	1.5	1.5	1.5	1.5	1.5	1.5	1.5
*c*_37_	150	300	150	150	150	150	150	150
*c*_38_	0.01	0.01	0.01	0.01	0.01	0.01	0.01	0.01
*c*_39_	0.5	0.5	0.5	0.5	0.5	0.5	0.5	0.5
*c*_40_	0.5	0.5	0.5	0.5	0.5	0.5	0.5	0.5
*c*_41_	0.8	0.8	0.8	0.8	0.8	0.8	0.8	0.8
*c*_42_	0.002	0.001	0.002	0.002	0.002	0.002	0.002	0.002
*c*_43_	2	2	2	2	2	2	2	2
*c*_44_	0.01	0.01	0.01	0.01	0.01	0.01	0.01	0.01
*c*_45_	0.5	0.5	0.5	0.5	0.5	0.5	0.5	0.5

##### Total carbon production

The total stemwood carbon production per hectare (TCP) at a certain stand age (*t*) can be described with equation (1) where TCP_*t*_ is the TCP at stand age *t*, TCP_*max*_ is the maximum TCP which will be reached at stand age *t*_*max*_ and *k* is a factor describing the shape of the increment curve.

$${\text{TCP}}_{t}={\text{TCP}}_{\text{max}}\cdot e^{k\cdot \overset{2}{\ln}(t/t_{\text{max}})}$$

This curve can be fitted to values from a yield table, field observations or model results. The curve fit allows estimating the maximum of the total carbon production and also the forest age, when this maximum is reached. The coefficient *k* allows describing how the increment is distributed over age. The estimated values may be different for different yield levels.

By knowing *k* and *t*_*max*_ the increment optimal harvesting time *t*_*opt*_ can be calculated with equation (2).

$$t_{\text{opt}}=t_{\text{max}}\times e^{0.5/k}$$

By dividing the TCP at time t_*opt*_ with t_*opt*_ the highest mean annual increment (MAI) can be calculated (equation 3). This MAI is used in the g4g-model to describe the yield level.

$$\text{MAI}=\frac{{\text{TCP}}_{t_{\text{opt}}}}{t_{\text{opt}}}$$

Typical TCP_*max*_, *k* and *t*_*max*_ are changing for different yield levels and so their values needs to be estimated depending on yield. The relation of MAI with the shape factor (*k*), the highest age until increment happens (*t*_*max*_) and maximum TCP (TCP_*max*_) are described with equation (4), 5 and 6.

$$k\;\;=\;\;c_{0}+c_{1}\times e^{c_{2}\times {\text{MAI}}^{c_{3}}}$$

$$t_{\text{max}}\;\;=\;\;c_{4}+\frac{c_{5}}{1+e^{\left(c_{6}+c_{7}\times \text{MAI}\right)}}$$

$${\text{TCP}}_{\text{max}}\;\;=\;\;\text{MAI}\times t_{\text{max}}\times e^{0.25/k}$$

##### Maximum stand density

The biomass of forests with low thinning (removal of dead trees) at a specific age is the TCP_*t*_ subtracted by the biomass of dead and removed trees until this age. The fraction of carbon in the living biomass (CMax_*t*_) compared to the TCP is described with equation (7).

$$\begin{array}{lll} \;\frac{{\text{CMax}}_{t}}{{\text{TCP}}_{t}}\;\; & =\;\;(cc_{0}+cc_{1}\times \ln(\frac{t}{t_{\text{opt}}}))\times {\left(1-cc_{2}\times \frac{t}{t_{\text{opt}}}\right)}^{c_{21}}\;\\ cc_{0}\;\; & =\;\;c_{8}+\frac{c_{9}}{1+e^{\left(c_{10}+c_{11}\times \text{MAI}\right)}}\; & \;\\ cc_{1}\;\; & =\;\;\frac{c_{12}}{1+e^{\left(c_{13}+c_{14}\times \text{MAI}\right)}}+\frac{c_{15}}{1+e^{\left(c_{16}+c_{17}\times \text{MAI}\right)}}\; & \;\\ cc_{2}\;\; & =\;\;c_{18}+c_{19}\times e^{\left(c_{20}\times \text{MAI}\right)}\; & \; \end{array}$$

The $cc_{2}\times \frac{t}{t_{\text{opt}}}$ defines the age where all the biomass of a forest has been died. If the estimated fraction of $\frac{{\text{CMax}}_{t}}{{\text{TCP}}_{t}}$ is lower than 0 it is set to 0 (age is beyond the age where all trees have died) and if it’s higher than 1 it is set to 1 (young stands where maximum density is not reached).

##### Managed stand density

The managed stand density depends on the thinning regime. Here the managed stand density is defined as the density where 95 % of the increment of a full stocked stand is produced. This point can be estimated using equation (12) describing the increment depending on stand density.

##### Tree size

The tree size has an influence on the harvesting costs and share of wood which can be used as sawn wood. The height development (*h*) over age (*t*) is described in equation (8)

$$h=c_{22}\times {\text{MAI}}^{c_{23}}\times {\left(1-e^{\left(c_{24}\times t\right)}\right)}^{c_{25}\times {\text{MAI}}^{c_{26}}}$$

Typical the height growth is not affected by the stand density as long as the tree does not belong to the suppressed trees which are here not considered. Different thinning methods (removing high trees versus removing small trees) can influence the average height, which is also not considered in this equation.

The calculation of the age until the height of 1.3 m is reached (*t*_*h*1.3_) can be done with equation (9).

$$t_{h1.3}=\frac{\ln(1-{\frac{1.3}{c_{22}\times {\text{MAI}}^{c_{23}}}}^{\frac{1}{c_{25}\times {\text{MAI}}^{c_{26}}}})}{c_{24}}$$

Until this age the diameter, which is typical measured at breast height (1.3 m above ground), is zero. At older ages the average diameter of a full stocked stand (*d*_*fs*_) will be calculated with equation (10).

$$\begin{array}{lll} d_{\text{fs}}\;\; & =\;\;cc_{3}\times {\left(1-e^{\left(cc_{4}\times (t-t_{h1.3})\right)}\right)}^{cc_{5}}\;\\ cc_{3}\;\; & =\;\;c_{27}+c_{28}\times \text{MAI}\; & \;\\ cc_{4}\;\; & =\;\;c_{29}/(1+c_{30}\times {\text{MAI}}^{c_{31}})\; & \;\\ cc_{5}\;\; & =\;\;c_{32}/(1+c_{33}\times {\text{MAI}}^{c_{34}})\; & \; \end{array}$$

##### Stand density dependency

Diameter and volume increment per hectare depend on stand density. The diameter estimation in equation (10) describes the diameter development over time at full stocked stands. At lower stocking degrees the DBH will be larger. Open grown trees have twice diameter compared to trees with the same age grown in full stocked stands [35]. Also thinning will influence the average diameter of a stand if smaller or larger trees are removed compared to those left in the stand [36] but these effects are not taken into consideration in this calculations. In the g4g–model the diameter of a tree grown permanently under other than full stocked stand densities (*sd*) can be calculated with equation (11) where *sd*=0 for open grown trees and *sd*=1 for full stocked stands.

$$\frac{d_{\text{sd}}}{d_{\text{fs}}}=2-sd^{c_{35}}$$

Also the increment per hectare depends on the actual stand density (*sd*). In the model this increment reaction is calculated with equation (12) which describes the relation of the increment at a specific stand density (*TCP*_*sd*_) and the increment at increment optimal stand density (*TCP*_*opt*_). The equation uses stand density (*sd*) yield (MAI) and forest age (*t*).

$$\begin{array}{lll} \frac{\text{TC}P_{\text{sd}}}{\text{TC}P_{\text{opt}}}\;\; & =\;\;\frac{cc_{11}-cc_{11}^{cc_{6}}}{cc_{9}}\;\\ cc_{6}\;\; & =\;\;1+\frac{t^{c_{36}}}{c_{37}}\times \frac{1}{c_{38}+c_{39}\times {\text{MAI}}^{c_{40}}}\; & \;\\ cc_{7}\;\; & =\;\;1+\frac{c_{41}}{1+c_{42}\times t^{c_{43}}\times \frac{1}{c_{44}+{\text{MAI}}^{c_{45}}}}\; & \;\\ cc_{8}\;\; & =\;\;{\left(\frac{1}{cc_{6}}\right)}^{\frac{1}{cc_{6}-1}}\; & \;\\ cc_{9}\;\; & =\;\;cc_{8}-cc_{8}^{cc_{6}}\; & \;\\ cc_{10}\;\; & =\;\;cc_{8}\times cc_{7}\; & \;\\ cc_{11}\;\; & =\;\;\left\{\begin{array}{ll} cc_{8} & \;\text{:}\;cc_{8}\leq \text{sd}\times cc_{10}\\ \text{sd}\times cc_{10} & \;\text{:}\;cc_{8}>\text{sd}\times cc_{10} \end{array}\right)\; & \; \end{array}$$

Both stand density adaptors work if the stand density stays constant over the whole rotation time. If the stand density is changing over time, these equations can also be used. But then is the question either to use the stand age or the actual tree size or something in between. The same is valid for the increment functions for changes in the yield. For the made calculations the stand age was used but with the current model structure it will also be possible to calculate a theoretical age of a tree e.g. by using the current tree height and calculating the years a tree will need under the current yield to reach this height like it was done in [37]. [8] describes sites where the increment in not full stocked stands is slightly higher than in full stocked stands. This effect is small and observed only on some sites with low productivity and is currently not reproduced by the model.

## Competing interests

The authors declare that they have no competing interest.

## Authors’ contributions

GK has made all calculations regarding the g4g–model and the results in this paper. He has also written the main part of the manuscript. SS has made the yield calculations with PICUS3G and has written the description of this calculations together with mjl and rs and reviewed the main article. TL has made the yield calculations with Prelued and has written the description of this calculations and reviewed the main article. AS has made the yield calculations with Gotilwa+ and has written the description of this calculations and reviewed the main article. WR has prepared and described the simulation database (soil, climate), implemented the PICUS3G model, and reviewed the main article. RS has developed and implemented the PICUS3G model, and has, together with mjl and ss, written the PICUS3G section. MJL has furthermore contributed to the design of the model linking approach and to design and implementation of the simulation data base of the three stand level models. All authors read and approved the final manuscript.
